# Demonstration of extrinsic chirality in self-assembled asymmetric plasmonic metasurfaces and nanohole arrays

**DOI:** 10.1038/s41598-024-68007-4

**Published:** 2024-07-26

**Authors:** Emilija Petronijevic, T. Cesca, C. Scian, G. Mattei, R. Li Voti, C. Sibilia, A. Belardini

**Affiliations:** 1 Department SBAI, Sapienza University of Roma, Via A. Scarpa 14, 00161 Rome, Italy; 2 Physics and Astronomy Department, University of Padova, Via Marzolo 8, 35131 Padova, Italy

**Keywords:** Splasmonics, Chirality, Nanomaterials, Metamaterials, Nanohole arrays, Optics and photonics, Metamaterials

## Abstract

Chirality, the lack of mirror symmetry, can be mimicked in nanophotonics and plasmonics by breaking the symmetry in light-nanostructure interaction. Here we report on versatile use of nanosphere lithography for the fabrication of low-cost metasurfaces, which exhibit broadband handedness- and angle-dependent extinction in the near-infrared range, thus offering extrinsic chiro-optical behavior. We measure wavelength and angle dependence of the extinction for four samples. Two samples are made of polystyrene nanospheres asymmetrically covered by silver and gold in one case and silver only in the other case, with a nanohole array at the bottom. The other two samples are nanohole arrays, obtained after the nanosphere removal from the first two samples. Rich extrinsic chiral features are governed by different chiro-optical mechanisms in the three-dimensional plasmonic semi-shells and planar nanohole arrays. We also measure Stokes parameters in the same wavelength and incidence angle range and show that the transmitted fields follow the extrinsic chirality features of the extinction dissymmetry. We further study the influences of the nanostructured shapes and in-plane orientations on the intrinsic vs extrinsic chirality. The nanoholes are modelled as oval shapes in metal, showing good agreement with the experiments. We thus confirm that nanosphere lithography can provide different geometries for chiral light manipulation at the nanoscale, with the possibility to extend functionalities with optimized oval shapes and combination of constituent metals.

## Introduction

Chirality at the nanoscale arises when there is a symmetry breaking in the nanostructure-light interaction^1–6^. A chiral nanostructured system differently interacts with circular polarizations of opposite handedness, leading to important consequences at both near- and far-field spatial scales. When left and right circular polarizations (LCP and RCP, respectively) are differently confined and enhanced in the nanostructures’ near-field^7^, regions of enhanced and handedness-dependent near-field chirality emerge. When this effect is applied to chiral substances in the nanostructures’ near-field, it shows great promise for ultra-sensitive chiral sensing^8,9^. Furthermore, chirality at the nanoscale was recently applied in surface enhanced Raman sensing^10–12^. In the far-field, chiral properties of the nanostructure in the linear regime can be revealed by exciting it with LCP and RCP and measuring differences in transmission^13,14^, absorption^15–17^ diffraction^18,19^ or reflection^20,21^. Moreover, second harmonic generation measurements can reveal chiral influence on nonlinear interactions^22,23^, while nanostructures can be designed to emit light with tailored circular polarization degree^24–26^. Therefore, chirality tailored by nanostructures is investigated by many scientific communities, with promising applications in multidisciplinary fields such as nanostructure-enhanced sensing, chiral emission and polarization manipulation to name a few.

With modern nanotechnology, nanostructures can be obtained in chiral shapes such as gammadion-like plasmonic arrays^27^, nano-helices^28,29^, nano-kirigamis^30^, periodic plasmonic chiral nanoholes^31^ etc. Furthermore, simple, self-assembling nanofabrication processes such as nanosphere lithography (NSL)^32–37^ can be combined with oblique angle deposition of metallic layers to obtain asymmetric or chiral nanostructures. In particular, metallic layers can be deposited in multiple steps (with different plasmonic flux directions) to obtain intrinsically chiral plasmonic shells on nanospheres^38,39^, nanocrescents^40,41^ and nanohole arrays^42^. However, nanostructure does not need to be chiral itself to produce chiral behavior: properly orienting the nanostructure with respect to the excitation/emission measurement set-up has been widely used to induce “extrinsic” chiral behavior in achiral nanostructures. For example, moon-like nanocrescents were shown to boost chiral behavior at large oblique angles in the near-infrared range^43^, while we previously used NSL to study asymmetric nanocrescents on dielectric nanospheres ^14,17–19,44^ and elliptical nanohole arrays (NHA)^45^. The latter are extremely simple planar plasmonic geometry, meant to be easily applied in sensing applications^46^. While the possibility of high intrinsic chirality in elliptical NHA is well understood^47,48^, their extrinsic features in self-assembled samples are not widely investigated, especially due to limitations concerning the uniformity of the nanohole geometry^49^.

In this work we first confirm the potentialities of NSL to obtain low-cost, asymmetric metasurfaces, which exhibit rich broadband chiro-optical response in the near-infrared range. By changing the geometrical parameters during NSL, we obtain four samples, which can be divided into two groups: metasurfaces made of polystyrene nanospheres (PSN) asymmetrically covered with a plasmonic layer containing a nanohole array at the bottom, and nanohole arrays (NHA), obtained by mechanically removing the PSN. For each group, we investigate one sample with a single silver layer, and one sample with layers of silver and gold. We perform extrinsic chirality experiment on these samples: in a broad near-infrared range, we measure the extinction dependence on wavelength, incident angle, and LCP/RCP. Next, we perform spectrally resolved full Stokes analysis of the output polarization state, which agrees with extinction circular dichroism. We then apply detailed modelling to investigate the origin of the chiro-optical behavior and focus on the oval nanohole geometry, which, for its simplicity, could easily find applications in emission or sensing.

## Results and discussion

### Broadband chiro-optical extinction

**Figure 1:**
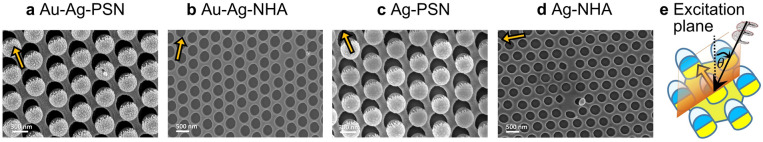
SEM images of the samples (**a**) Au–Ag–PSN, (**b**) Au–Ag–NHA, (**c**) Ag–PSN, and (**d**) Ag–NHA. (**e**) Sketch of the excitation plane. Yellow arrow indicates projection of the metal flux direction onto the sample surface plane.

Samples were produced by NSL on soda-lime glass substrates, as detailed in Sect “Methods”. We investigate four samples, the details of which are given in Table 1. Scanning electron microscopy (SEM) images of the samples are shown in Fig. 1: all starting PSN samples were covered by plasmonic layers by using tilted metal evaporation at 45°. Yellow arrow indicates projection of the metal flux direction onto the sample surface plane. The first sample, Au–Ag–PSN, is a metasurface based on PSN asymmetrically covered with 9 nm of Ag and a 43 nm of Au; due to the shadowing effect of the tilted metal flux, NHA on the bottom is created, Fig. 1a. Sample Au–Ag–NHA was obtained by mechanically removing PSN from Au–Ag–PSN, resulting in an array of nanoholes, Fig. 1b. Sample Ag-PSN was obtained by evaporating 55 nm of Ag on self-assembled PSN, Fig. 1c. Finally, sample Ag–NHA was obtained by removing PSN from Ag–PSN, as shown in Fig. 1d.

**Table 1:** Nanofabrication parameters of the four samples.

Au–Ag–PSN	9 nm of Ag + 43 nm of Au
Au–Ag–NHA	9 nm of Ag + 43 nm of Au
Ag–PSN	55 nm of Ag
Ag–NHA	55 nm of Ag

We first spectrally characterize our samples by measuring their extinction with a set-up used in Ref.^14^, in the range of 700–1000 nm (see Sect “Methods”). We orientate each sample so that it follows the extrinsic chirality rule: the light wave-vector, the vector perpendicular to the sample’s surface, and the vector pointing in the metal flux direction must form a non-planar triad of vectors. Therefore, each sample is oriented so that its “yellow arrow” lies perpendicular to the incidence plane, as sketched in Fig. 1e. When excitation beam arrives at non-zero angle of incidence *θ*, dissymmetry in interactions with LCP and RCP can be expected.

**Figure 2:**
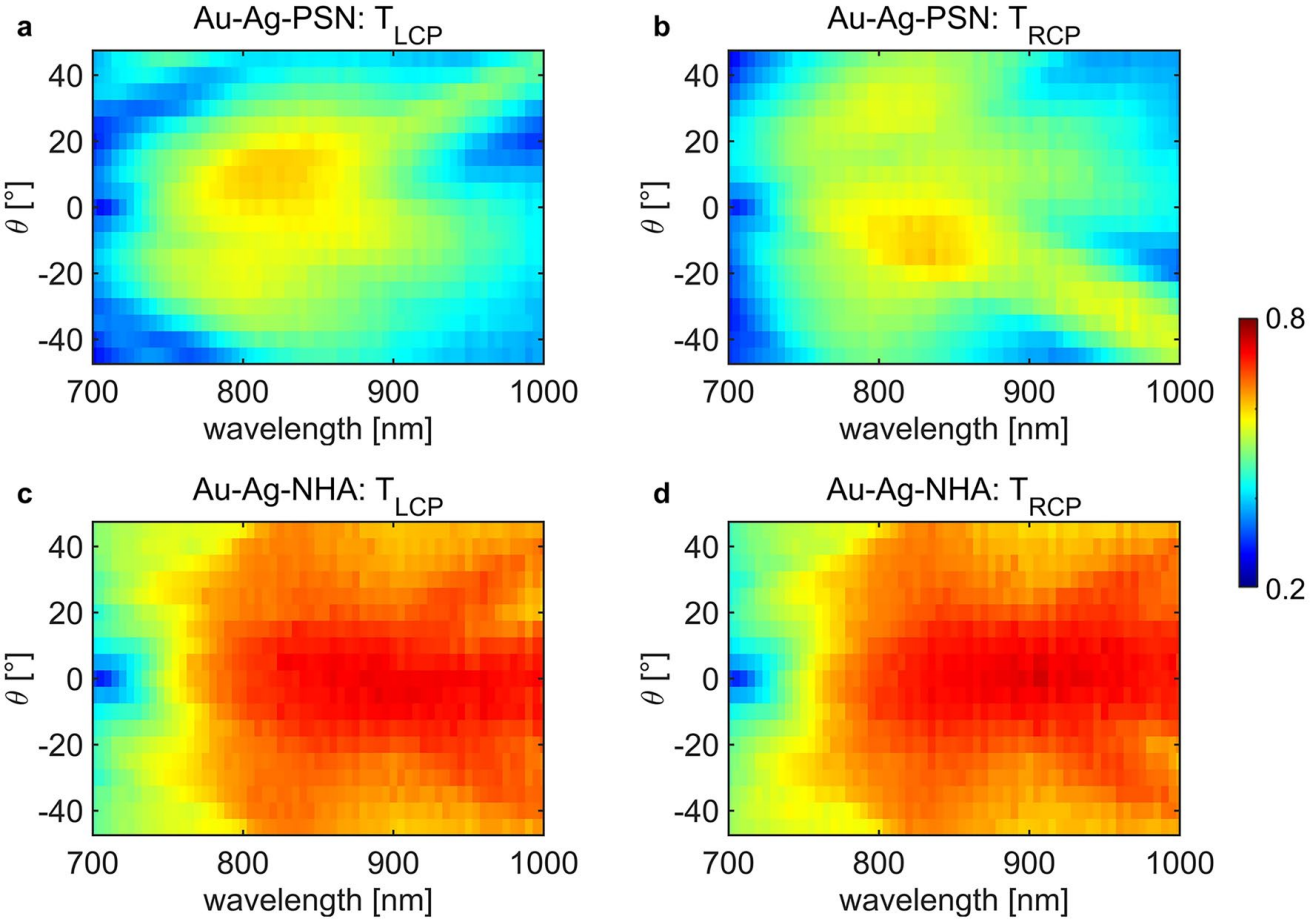
Wavelength-incidence angle (*θ*) transmission maps for Au–Ag–PSN excited with (**a**) LCP and (**b**) RCP, and for Au–Ag–NHA excited with (**c**) LCP and (**d**) RCP.

Firstly, we show measured wavelength-*θ* transmission maps for LCP and RCP excitations, denoted as T_LCP_ and T_RCP_, respectively. The dispersion maps for Au–Ag–PSN in Fig. 2a and Fig. 2b show complex resonant features: broadband, near-infrared transmission reaching 0.6 is intersected by zones of transmission minima, with linear dependence on wavelength λ. This corresponds to extinction maxima that we noticed in Au-based metasurfaces in our previous work^14^. Low transmission zones invert for LCP and RCP excitation with inversion of *θ*. Linear dependence on wavelength and handedness inversion behavior with respect to *θ* show that the extrinsic chirality in Au–Ag–PSN is governed by both the metasurface periodicity and the asymmetric shapes of the titled hybrid plasmonic shells and NHA.

As expected, removing the PSN leads to higher transmission in Au–Ag–NHA, as shown in Fig. 2c, d. Here, the difference between T_LCP_ and T_RCP_ maps is highly diminished. However, we can see that in the 950–1000 nm wavelength range, LCP excitation gives higher transmission around *θ* =  − 20° than around *θ* = 20°, and this behavior is exactly inverted for RCP excitation. In general, this sample exhibits broad zones of high near-infrared transmission for both LCP and RCP excitations, intercepted with additional features present of lower transmission, similar to the ones present in Au–Ag–PSN. T_LCP_ and T_RCP_ are completely anti-symmetric with respect to normal incidence, which is a typical behavior of extrinsic chirality.

**Figure 3:**
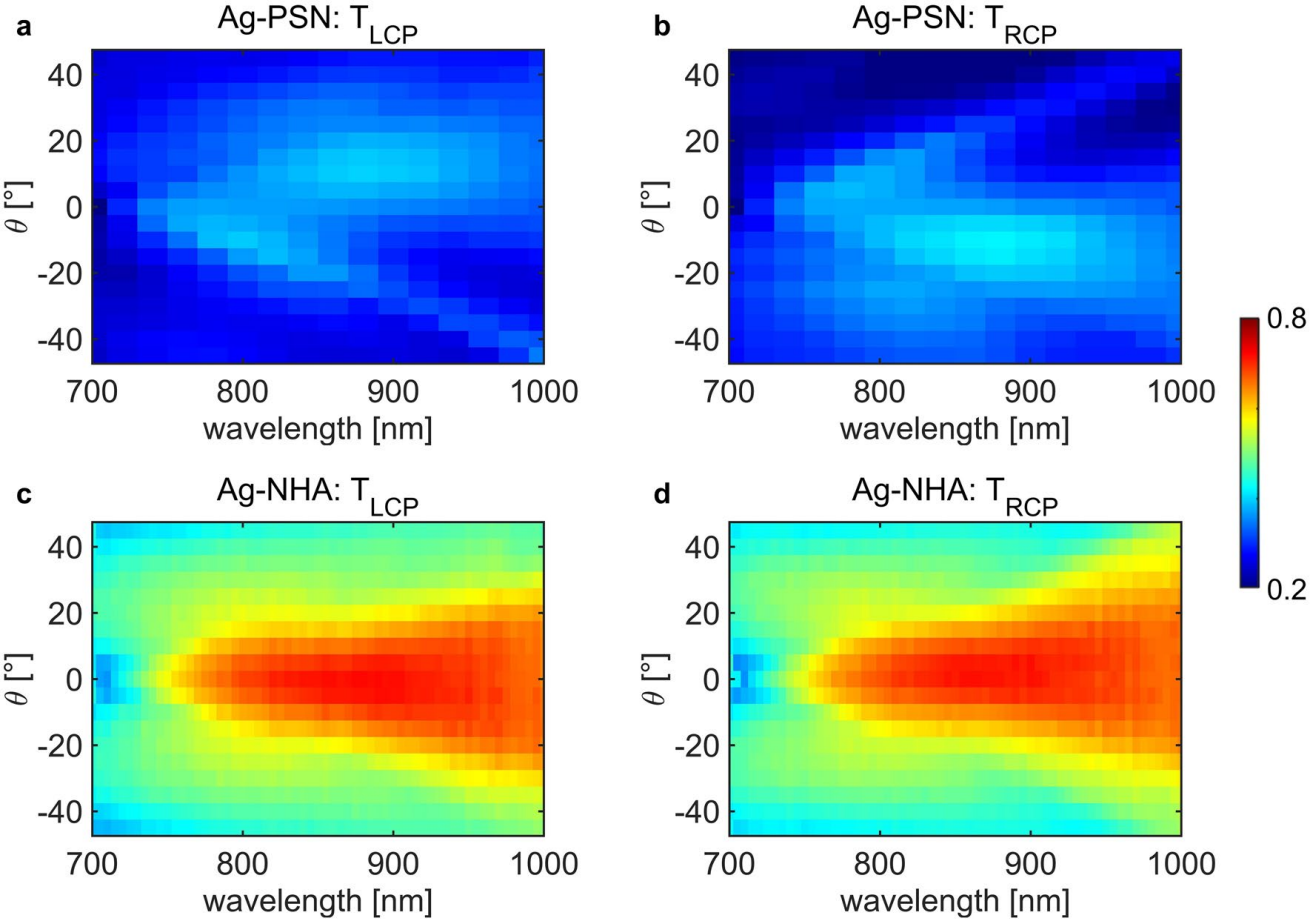
Wavelength- incidence angle (*θ*) transmission maps for Ag–PSN excited with (**a**) LCP and (**b**) RCP, and for Ag–NHA excited with (**c**) LCP and (**d**) RCP.

Next, we perform the same characterization of samples that were covered with only one plasmonic layer—55 nm of silver. For both Ag–PSN (Fig. 3a, b), and Ag–NHA (Fig. 3c, d), the total transmitted signal is significantly lower than in the sample with Au. Moreover, Ag–PSN is perfectly antisymmetric with respect to the normal incidence for LCP and RCP excitations, while in Ag–NHA, these polarizations get transmitted in a seemingly equal way. We note that a thin layer of Ag is frequently used as an adhesion layer for Au deposition on dielectric nanostructured templates; this way, one can use near-field electromagnetic field enhancements due to excellent plasmonic properties of Au in the near-infrared range. Using Ag can instead blue shift the resonant behavior of NHA^48^. We notice that low transmission features in Fig. 3a, b are blue-shifted with respect to the ones in Au–Ag–PSN. Moreover, Ag is more prone to oxidation and surface deterioration over time.

In the following, we define the zeroth order extinction as Ext_LCP,RCP_ = 1 − T_LCP,RCP_, and calculate the extinction dissymmetry factor g_ext_ as:

1 $${\text{g}}_{{{\text{ext}}}} \left[ {\text{\% }} \right] = \frac{{100\left( {{\text{Ext}}_{{{\text{LCP}}}} - {\text{Ext}}_{{{\text{RCP}}}} } \right)}}{{{\text{Ext}}_{{{\text{LCP}}}} + {\text{Ext}}_{{{\text{RCP}}}} }}.$$

**Figure 4:**
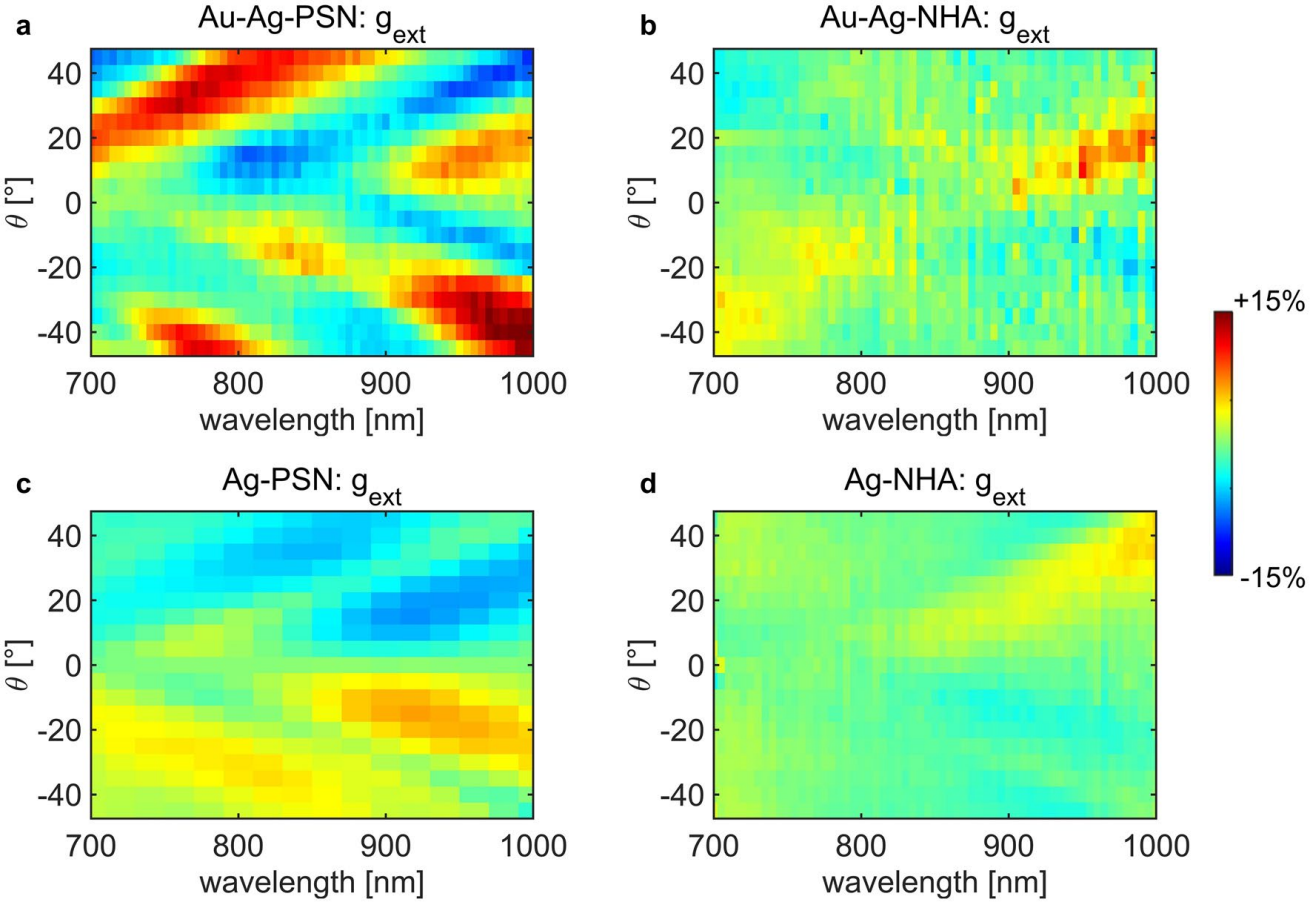
Experimental wavelength- incidence angle (*θ*) extinction dissymmetry g_ext_ maps for Samples (**a**) Au–Ag–PSN, (**b**) Au–Ag–NHA, (**c**) Ag–PSN, and (**d**) Ag–NHA.

The results are shown in Fig. 4. Sample Au–Ag-PSN shows complex and rich resonant behavior with absolute g_ext_ reaching 15% for large wavelength and *θ*. In extrinsic chirality experiments, g_ext_ should be equal to zero at normal incidence, and it should be anti-symmetric with respect to *θ*. In our previous work^44^, we characterized absorption CD at a single wavelength in similar samples. We showed that a non-zero CD at normal incidence, and deviation of anti-symmetric features, arise as a consequence of the additional in-plane tilt during the NSL. In the present case (Fig. 4a), this is even more evidenced in a broad wavelength range. In Fig. 4b, Au–Ag–NHA shows low absolute g_ext_ (average ~ 4%), with extrinsically chiral features which follow the dispersion lines of increased extinction in Fig. 2(b). In Fig. 4(c), Ag-PSN leads to much lower g_ext_ than Au–Ag–PSN, while they perfectly match the sign inversion due to the extrinsic chirality. Finally, a very weak extrinsic chiral behavior is also present in Ag–NHA (Fig. 4d).

### Stokes parameter control

**Figure 5:**
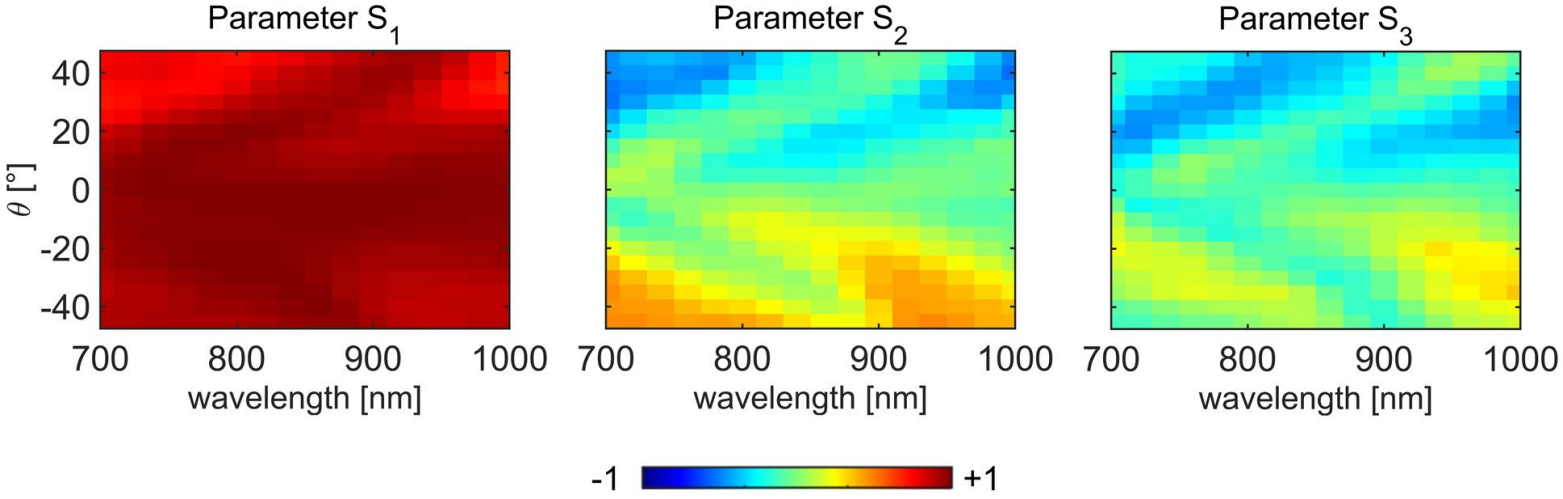
Normalized Stokes parameters of the transmitted field when Ag–PSN is excited with linear p-polarized light.

We next perform experimental full Stokes parameters characterization for sample Ag–PSN, as it gave perfect extrinsic chiral behavior in extinction. We excite this sample with linear polarization and place a quarter wave plate and a linear polarizer before the photodiode. For each wavelength and incidence angle, four combinations of QWP-LP orientations are used to resolve S0, S1, S2 and S3, as explained in the Supplementary Material. We normalize S1, S2 and S3 to S0, and plot the resulting maps in Fig. 5. The normalized Stokes parameters S_1_ and S_2_ show the prevalence of linear polarizations: horizontal with respect to vertical, and diagonal + 45° with respect to -45°, respectively; without the sample, p-polarized excitation corresponds to S_1_ = 1 (horizontal). More importantly, S_3_ describes the dominance of RCP with respect to LCP, and in Fig. 5 it is evident that it closely follows the g_ext_ behavior from Fig. 4c. This is in agreement with our recent findings in samples based on asymmetric nanoshells (without NHA). It proves that, in general, the origin of chiro-optical effects lies in the symmetry breaking of the interaction between LCP and RCP excitation with the nanostructure: when the nanostructure is excited with linear polarization at oblique incidence within a configuration of non-planar triad of vectors, the transmitted polarization state carries circular polarization degree according to the rules of extrinsic chirality (i.e. handedness inversion with *θ*).

### Numerical investigation

**Figure 6:**
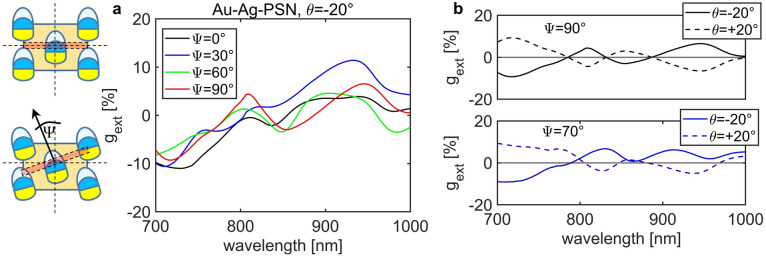
Simulated influence of the in-plane tilt angle Ψ on g_ext_. Inset: the tilt definition w.r.t. incidence plane (red line, perpendicular to the surface). (**a**) Au–Ag–PSN behaviour of g_ext_ , at *θ* =  − 20° changes with the in-plane tilt Ψ. (**b**) The tilt angle of Ψ = 70° fits well the non-perfect extrinsic chirality features at *θ* =  − 20°.

To understand the origin and behavior of g_ext_ in different samples, we model the electromagnetic behavior of the samples by a commercial 3D finite-difference time domain (FDTD) solver by Lumerical^50^ (see Sect “Methods” and Supplementary Material). Firstly, we aim to explain the “non-extrinsic” behavior of g_ext_ in Au–Ag–PSN (map in Fig. 4a). Namely, especially at large incidence angles *θ*, switching from *θ* to − *θ* leads only to the sign inversion of g_ext_, while its absolute values differ (e.g. note the branches from 700 to 800 nm for |*θ*|> 20°). Moreover, this sample, when excited at normal incidence, has a low negative g_ext_ around 900 nm, suggesting the presence of intrinsic chirality. The origin of this effect lies in the misalignment between the metal flux direction projected on the sample surface (yellow arrow in Fig. 1) and the triangular symmetry of the lattice. Namely, coupling of the incoming light to the modes of periodically arranged nanostructures is governed not only by the wave-vector matching conditions and the lattice rotational symmetry^51,52^, but also by the symmetry of the nanostructured shape^53^. In our case, the plane of incidence is always perpendicular to the flux projection, as sketched by the reddish line in the inset of Fig. 6; however, the flux projection can be misaligned with the triangular symmetry by the azimuthal angle Ψ. For Ψ = 0° and Ψ = 90°, different lattice modes (as defined by Brillouin zone) would lead to “perfect” extrinsic chirality. We therefore investigate the influence of Ψ on g_ext_ for *θ* =  − 20° in the sample Au–Ag–PSN.

Figure 6a shows that, for periodically organized 3D shapes such as plasmonic semi-shells, g_ext_ is not drastically influenced by the in-plane tilt: there is always a negative chirality for shorter wavelengths, which changes the sign to positive, crossing zero in the 780–820 nm range. The oscillatory behavior of g_ext_ is typical of diffractive metasurfaces, as we previously showed also with different optothermal techniques^15,17^. However, what leads to the non-anti-symmetric features is the Ψ tilt from the symmetry lines, as shown in Fig. 6b: the best fit to the experimental data is observed for Ψ = 70°. This effect is kept when the nanospheres are removed, as demonstrated in the map of Au–Ag–NHA, Fig. 4b: in the 900–1000 nm range, g_ext_ follows higher intensity for positive than for negative angles. On the other hand, the only Ag-based sample, with and without the nanospheres, shows almost perfect extrinsic behavior, meaning that the in-plane offset is negligible.

**Figure 7:**
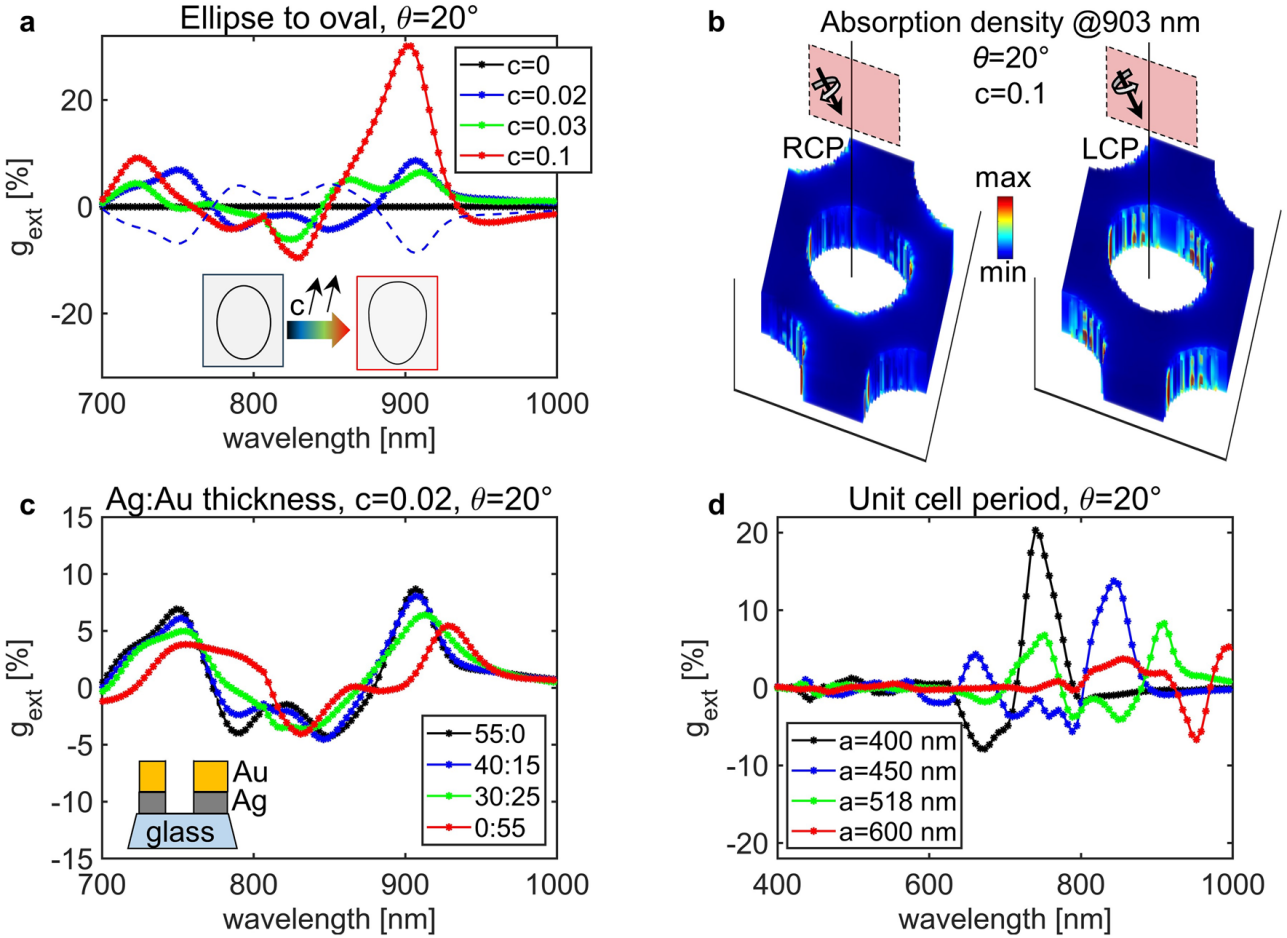
(**a**) g_ext_ of oval nanohole arrays depends on the shape factor c; presented results are for Ag–NHA at *θ* = 20°. The best fit to the experimental data is obtained with c = 0.02 (dashed lines are the inversion of g_ext_ with *θ* =  − 20°). (**b**) Absorption density for strongly oval shape (c = 0.1), excited at 903 nm and *θ* = 20° strongly depends on the excitation handedness. (**c**) Adding Au on the top of Ag red-shifts the resonances: the total thickness is 55 nm, while legend presents Ag to Au thickness ratio. (**d**) g_ext_ spectral position is defined by the grating nature of the NHA; at a single angle of incidence *θ* = 20°, increasing the starting interparticle distance red shifts the g_ext_ peak.

We next focus on the NHA geometry, which is of interest as it could be a simple plasmonic substrate for sensing and emission applications ^51,52,54^. In principle, the Ψ tilting of the elliptically shaped nanoholes was predicted to lead to chirality at normal incidence^47^; however, the SEM images of Ag–Au–NHA and Ag–NHA show that, if nanoholes in our samples were modelled as tilted ellipses, the short and long diameters would not differ enough to produce considerable intrinsic chirality (at *θ* = 0°). Moreover, the elliptical shape model could not explain the behavior at oblique incidence. Due to the shadowing effect, the nanoholes have a slightly oval, rather than elliptical shape. To decouple intrinsic from extrinsic chirality, we study the effect of such oval nanohole shape in Ag–NHA. We model the nanohole with “egg” shape etched in 55 nm of Ag and defined by the parameter c and the nanohole’s “longest diameter” ${D}_{tot}$, as sketched in the inset of Fig. 7a (the simulation details are given in the Supplementary Material). In Fig. 7a, we show how c influences simulations of g_ext_ for Ag–NHA excited at *θ* = 20°. When the shape is purely elliptical (c = 0), g_ext_ is 0° at oblique incidence. Increasing c leads to the symmetry breaking and different interactions between LCP and RCP, as it can be seen in different distributions of the electric field intensity in the unit cell, Fig. S1; g_ext_ exhibits the oscillatory behavior, with the peak around 900 nm reaching 30% for c = 0.1. Under this condition, the coupling with LCP and RCP strongly differs in the oval shape; we therefore visualize the absorption density over the unit cell in Fig. 7b. The best fit regarding the absolute value of the measured g_ext_ is for c = 0.02 (dashed lines show that g_ext_ at *θ* =  − 20° simply inverts sign). We further keep c = 0.02 and study the influence of the plasmonic material thickness and type; if the nanoholes are in 55 nm of Au instead of Ag, the g_ext_ main peak red-shifts for ~ 30 nm, Fig. 7c. Therefore, using the plasmonic properties of Ag, and covering it with a thin layer of Au to prevent the oxidation will not change the main aspects of extrinsic chiral coupling with oval shapes. Other parameters can be tuned to enhance g_ext_ or position it in a different spectral range. For example, the starting PSN diameter defines the periodicity of the array, which positions the branches of increased extinction with increasing incidence angle, with different coupling to LCP and RCP due to the extrinsic chirality. This is shown in Fig. 7d for Ag-NHA with smaller and larger periodicities (other geometric parameters are scaled w.r.t. a = 518 nm).

Very recently, nanoholes with oval shapes, organized in quadratic unit cells, were predicted to exhibit remarkable chiral properties governed by the excitation of quasi bound state in a continuum^55^: they led to almost 90% of circular dichroism in the near-infrared absorption. Interestingly, our extinction experiments show similar trends of g_ext_ w.r.t. angle of incidence, although with very low efficiency and Q factors. We believe that NSL can be further explored to study modes in oval nanoholes organized in hcp cells, e.g. by optimizing small evaporation angles to reduce the linear birefringence.

## Conclusion

Our results confirm versatile near-infrared extrinsic chirality in samples obtained by nanosphere lithography and provide new insights for the use of simple geometries such as NHA. We characterized asymmetric metasurfaces and NHA, fabricated with a single Ag layer, or with Ag and Au layers. The dissymmetry factor g_ext_ can be tuned in broad wavelength and incidence angle ranges, while it also depends on the in-plane tilt of the nanostructured shape. In uniform samples aligned with hexagonal symmetry lines, a perfect inversion of g_ext_ with the inversion of the incidence angle is expected. We first measured this behavior in the metasurface based on Ag; we further resolved its Stokes parameters, and proved that S_3_ exhibits similar map with g_ext_. Extensive numerical modelling helps in understanding the differences between the g_ext_ maps and decoupling intrinsic from extrinsic chiro-optical effects. Moreover, a new way to model the NHA shape as oval rather than elliptical allows for good agreement with the experiment and leaves room for further improvements of the fabrication parameters. Finally, we believe that this work could open new ideas for low-cost flat plasmonic metasurfaces, where only 50 nm of plasmonic layer actively leads to tunable chiro-optical behavior in the visible and near-infrared range.

## Methods

### Fabrication of the metasurfaces and nanohole arrays

The first step for all four samples is the self-assembling of commercial polystyrene nanoparticles (PSN, MicroParticles GmbH, Germany) into a close-packed monolayer on a soda-lime glass substrate. The starting diameter of the PSN, which defines the final pitch a of the metasurfaces’ hexagonal unit cell, was *a* = 518 nm for all the samples. Then, we applied reactive ion etching to reduce the PSN diameter (to about Dp = 340–360 nm), while preserving the PSN 2D ordered arrangement. Next, for Au–Ag–PSN metasurface, a thin (9 nm) layer of Ag, and a 43 nm layer of Au were thermally evaporated on the PSN array, at 45° with respect to the surface’s normal; this resulted in the formation of asymmetric plasmonic shells on the top of the PSN, and of a nanohole array (NHA) on the substrate (due to the shadow effect). Au–Ag–NHA was then obtained by mechanically removing the PSN. The silver-based pair of samples (Ag–PSN and Ag–NHA) were obtained following a similar process, with 55 nm of Ag only.

### Extrinsic chirality measurements

We use a widely tunable (700–1000 nm) laser Chameleon Ultra II (Coherent Inc., Santa Clara, CA, USA) in linear mode, by modulating its output by a mechanical chopper at 70 Hz. We create LCP and RCP state by putting a linear polarizer (LP) and a quarter wave plate (QWP) on the light path, and rotating the QWP by − 45° and 45°, respectively. Such beam is impinging at an angle *θ* with respect to the surface normal, from air onto the patterned side of the samples. Total transmission is detected by a photodiode on the pathway of the fundamental order. All measurements are done at room temperature, and the transmission is normalized to the signal without the sample. To resolve transmitted polarization state in Ag–PSN, we performed Stokes parameters analysis (see Supplementary material).

### Numerical simulations

Extensive full-wave electromagnetic 3D simulations were performed by using the finite-difference-time-domain (FDTD) module in Lumerical. The simulation domain was defined as a periodically repeated unit cell of *a*√3•*a* area, with Bloch boundary conditions in x- and y- directions, and perfectly matching layers in the z-direction (*a* = 518 nm). The metasurface which models samples Au–Ag–PSN and Ag–PSN consists of polystyrene nanospheres covered by asymmetric shells, and standing on a semi-infinite glass substrate; we followed material parameters of Table 1, and used ellipsometry data for Ag and Au. To study the in-plane chirality, the shell is tilted around the vertical axis for angle Ψ. The metasurface was excited from the top (air) side, with a broadband plane-wave source. Since we investigate oblique incidence up to 45°, we used a broadband fixed angle source technique (BFAST). Therefore, two BFAST sources with properly shifted phases model circular polarizations. The incidence plane was defined as in the experiment, meaning that both BFAST sources were tilted in azimuthal direction for the same angle Ψ. The FDTD region was meshed with an accuracy of 4. To model the NHA, we considered oval shapes, with the details given in the Supplementary material. As in the experiment, we detected a zeroth order transmission by a transmission power monitor.

### Supplementary Information

Supplementary Information.

## Data Availability

The datasets used and/or analysed during the current study available from the corresponding author on reasonable request.
