# Supplementary material for: Demonstration of extrinsic chirality in self-assembled asymmetric plasmonic metasurfaces and nanohole arrays
Source: Sci Rep. 2024 Jul 26;14:17210. doi: 10.1038/s41598-024-68007-4 (PMC11282274; doi:10.1038/s41598-024-68007-4)
Supplement: Supplementary file 1 — Supplementary Information. [file 41598_2024_68007_MOESM1_ESM.docx]

**Demonstration of extrinsic chirality in self-assembled asymmetric plasmonic metasurfaces and nanohole arrays**

**SUPPLEMENTARY MATERIAL**

Emilija Petronijevic^†*^, T. Cesca^‡^, C. Scian^‡^, G. Mattei^‡^, R. Li Voti^†^, C. Sibilia^†^ and A. Belardini^†^

†Sapienza University of Rome, SBAI Department, Via Antonio Scarpa 16, 00161 Rome, Italy

‡Physics and Astronomy Department, University of Padova, via Marzolo 8, I-35131 Padova, Italy

**1. Stokes parameters measurements**

To study the Stokes parameters in a broad wavelength-incidence angle range, the Ag-PSN metasurface was excited from the nanostructured side with a p-polarized light. To resolve the transmitted polarization, a quarter wave plate (QWP) and a linear polarizer (LP) were put into the beam path, after the sample, but before the PD, and four combinations of their orientations were measured. We define φ_QWP_ as the QWP fast axis angle w.r.t. vertical axis, while φ_LP_ is the LP angle w.r.t. horizontal axis. In the first three measurement, φ_QWP_ is fixed at 0°, while the LP acquires angles φ_LP_=0° (horizontal), φ_LP_=45° (diagonal) and φ_LP_=90° (vertical); these three measurements give intensities I_1_, I_2_ and I_3_, respectively. In the fourth measurement, I_4_ gives the transmitted intensity for φ_QWP_=φ_LP_=45°. Finally, Stokes parameters are obtained as: S0=I_1_+I_3_, S1=I_1_-I_3_, S2=S0-2I_4_, S3=2I_2_-S0. Parameters S_1_, S_2_ and S_3_ in the main manuscript are obtained by dividing parameters S1, S2 and S3 by S0.

**2. Numerical simulations**

We use the modelling approach applied for similar samples in refs. [1,2], with nanoshell dimensions estimated from the SEM images and fabrication parameters. Dimensions of the FDTD domain in the xy plane are $a$ and $a\surd3$, where$a$ is the starting polystyrene nanosphere (PSN) diameter; this plane is surrounded by Bloch boundary conditions, while the z-direction is closed by the Perfectly Matched Layers in the air and in the glass. PSNs are modelled as dielectric spheres of diameter $D_{p}$ and refractive index n_PSN_ =1.61, standing on a glass substrate (n_glass_=1.51). The dielectric properties of the Au and Ag layers are obtained from ellipsometric measurements of thin films on the glass substrate deposited with the same evaporation conditions as for the samples. The metasurface is excited by two perpendicular BFAST sources which differ only in phase: the phase difference of 90° (-90°) models RCP (LCP) illumination.

We model the oval nanoholes by using the following equation for the “egg”-like planar shape:

$$x=\frac{D_{long}}{2}\cos\left( \varphi\right), y=\left( p-c \right)\cdot\frac{D_{long}}{2}+c\cdot\frac{D_{long}}{2}\cdot\cos\left( \varphi\right)\cdot\sin\left( \varphi\right),$$

where $D_{long}$ is the longest diameter, $c$ is the shape factor, $p$ is the ratio between the shorter diameter and $D_{long}$, and $\varphi$ varies over $2\pi$. To investigate the influence of intrinsic chirality, the sources, the plasmonic semi-shells and the nanoholes are rotated to reproduce the configuration in the inset of Fig. 6.

To study the influence of the shape factor on g_ext_ in Ag-NHA (Fig. 7(a)), parameters $D_{long}=390nm$ and $p=360/390$ were estimated from the SEM image and fabrication parameters. The unit cell distributions of the absorption density in Fig. 7(b) were calculated for $D_{long}=390nm$, $p=0.92$, and $c=0.1$ at 903 nm; these are parameters of the maximum g_ext_ in Fig. 7(a). To study the influence of the material combination (Fig. 7(c)), we kept the following parameters constant: $D_{long}=390nm$, $p=360/390$ and $c=0.02$ and changed the thicknesses of the metals: e.g. 30:25 corresponds to the simulation in which 30 nm of Ag was covered by 25 nm of Au. To study the influence of the metasurface periodicity on the spectral features of g_ext_ (Fig. 7(d)), we linearly scale all other dimensions in the model (PSN and semi-shell diameters and the oval shape diameters) with the factor a[nm]/518.


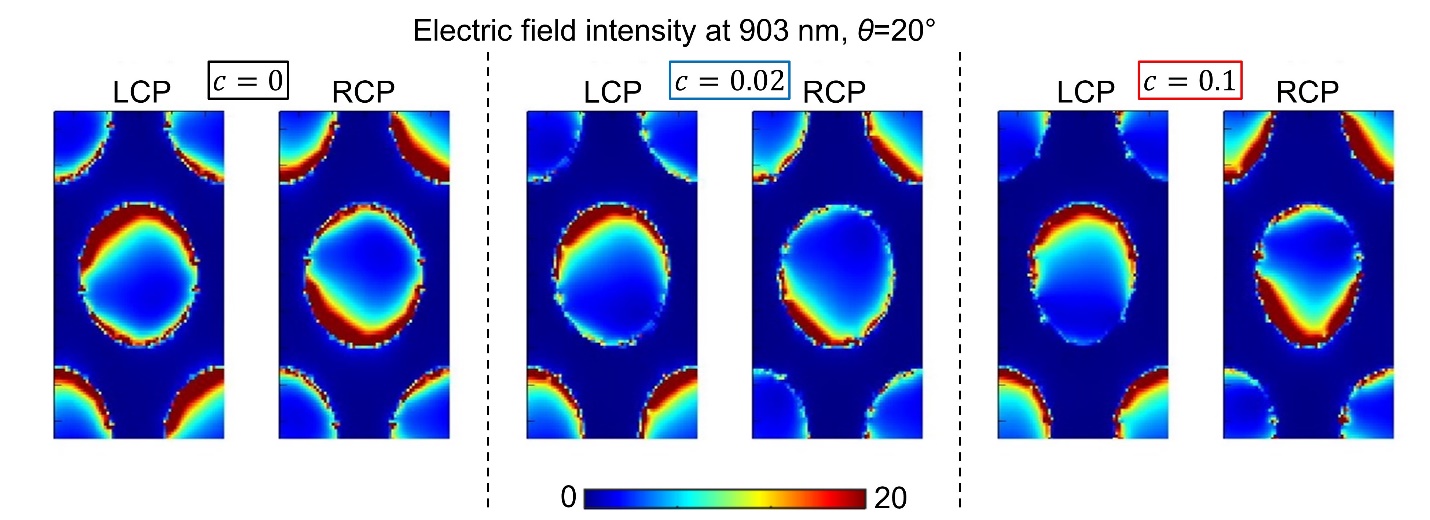


Figure S1. Electric field intensity for Ag-NHA excited at 903 nm and at *θ*=20°, monitored across the unit cell at 10 nm above the substrate. Nanohole shape parameters kept constant are $D_{long}=390nm$, $p=0.92$, while the increase of the oval shape parameter $c$ leads to the increased difference in coupling with LCP and RCP. The excitation wavelength was chosen from the g_ext_ maximum in Fig. 7(a).

**References**

^1^Petronijevic, E. et al. Photo-acoustic detection of chirality in metal-polystyrene metasurfaces. Appl. Phys. Lett. 114, 053101 (2019).

^2^Petronijevic, E., Belardini, A., Cesca, T., Scian, C., Mattei, G. & Sibilia, C. Rich near-infrared chiral behavior in diffractive metasurfaces. Phys. Rev. Appl. 16(1), 014003 (2021).
